# Supplementary material for: Influence of AAV vector tropism on long-term expression and Fc-γ receptor binding of an antibody targeting SARS-CoV-2
Source: Commun Biol. 2024 Jul 16;7:865. doi: 10.1038/s42003-024-06529-3 (PMC11250830; doi:10.1038/s42003-024-06529-3)
Supplement: Supplementary file 2 — Supplementary Information [file 42003_2024_6529_MOESM2_ESM.pdf]

Supplementary Information

Influence of AAV vector tropism on long-term expression and Fc-γ receptor binding of an antibody targeting SARS-CoV-2

Jannik T. Wagner[1*], Sandra M. Müller-Schmucker[1], Wenjun Wang[2], Philipp Arnold[3], Nadja Uhlig[4], Leila Issmail[4], Valentina Eberlein[4], Dominik Damm[1], Kaveh Roshanbinfar[5], Armin Ensser[1], Friederike Oltmanns[1], Antonia Sophia Peter[1], Vladimir Temchura[1], Silke Schrödel[6], Felix B. Engel[5], Christian Thirion[6], Thomas Grunwald[4], Manfred Wuhrer[2], Dirk Grimm[7], Klaus Überla[1*]

Inventory of supplementary information

Figure S1:	Uncropped blot images
Figure S2:	Transmission electron microscopic (TEM) visualisation of AAV vector particles
Figure S3:	TRES6 serum concentrations in challenged animals
Figure S4:	Anti-drug T cell responses
Figure S5:	Murine IgG2c Fc-glycosylation of AAV8 encoded antibodies
Figure S6:	Murine IgG2c Fc-glycosylation of AAVMYO encoded antibodies
Figure S7:	Changes in Fc-γ receptor binding over time
Table S1:	Transcriptomic expression analysis of genes associated with glycosylation pathways
Figure S8:	Gating strategy for flow cytometry based antibody-binding assays
Figure S9:	Gating strategy for the analysis of anti-drug T cell responses

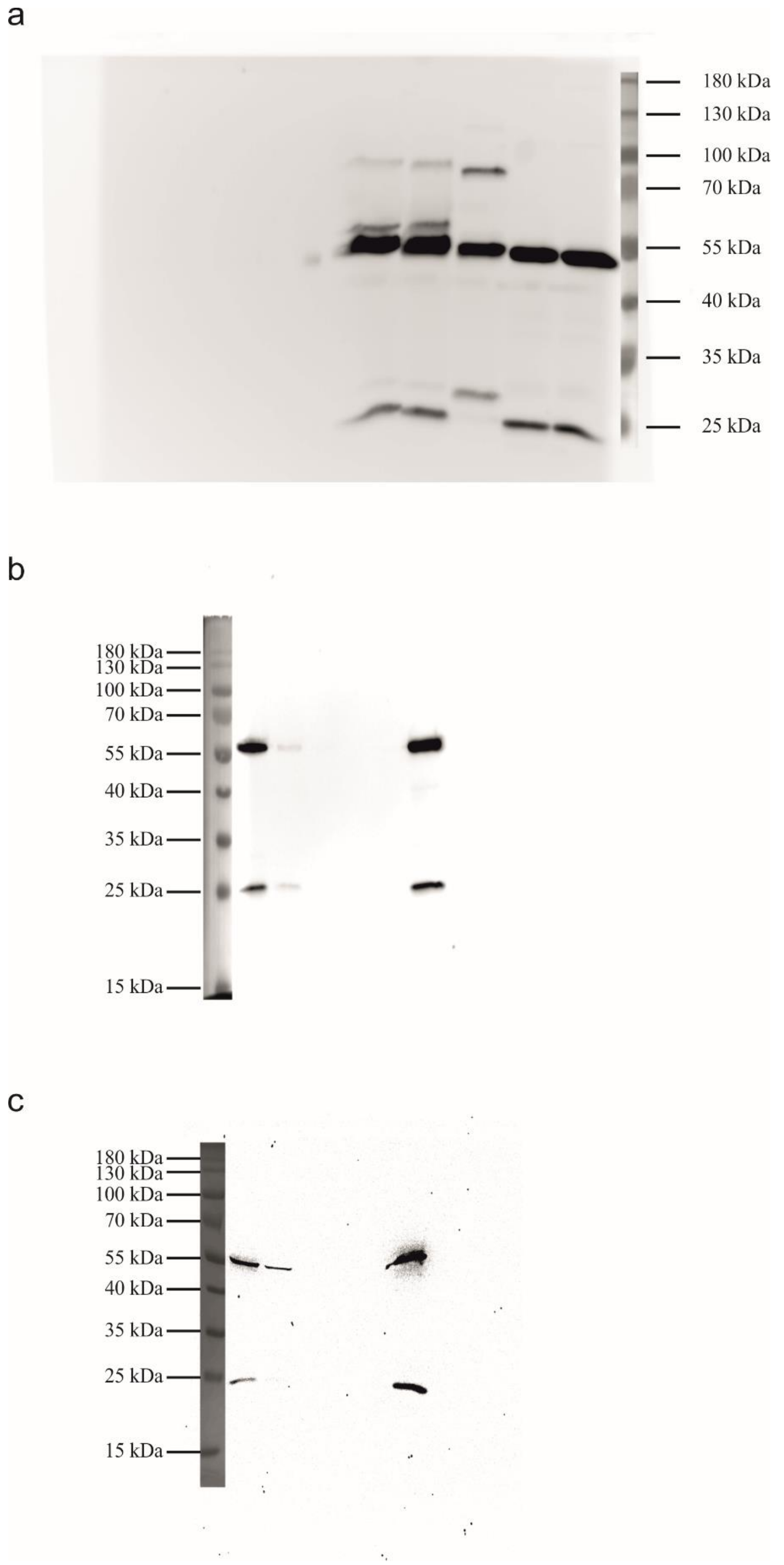

Figure S1 Uncropped blot images. *Shown are the uncropped western blot images of Fig. 2a (a), Fig. 2c (b) and Fig. 2d (c).*

A **B**

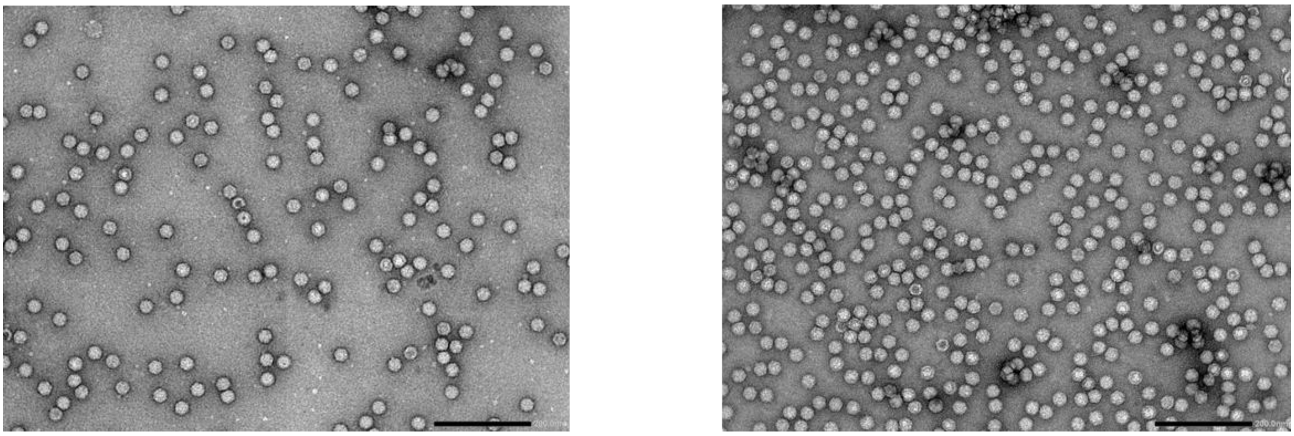

Figure S2. Transmission electron microscopic (TEM) visualisation of AAV vector particles. *AAV8 (A) and AAVMYO (B) vector particles were imaged by negative stain transmission electron microscopy. The scale bar represents 200 nm.*

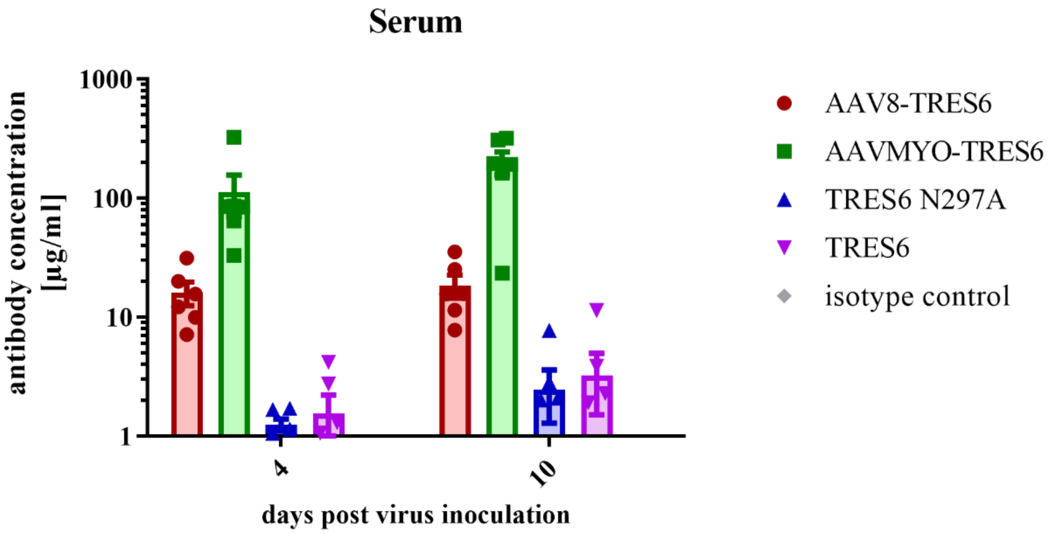

Figure S3. TRES6 serum concentrations in challenged animals. *The antibody concertation was determined in the sera of the challenged animals (n=6, ♀ = 6) on the indicated days after virus inoculation. The bars represent the mean antibody concentration of each group +SEM.*

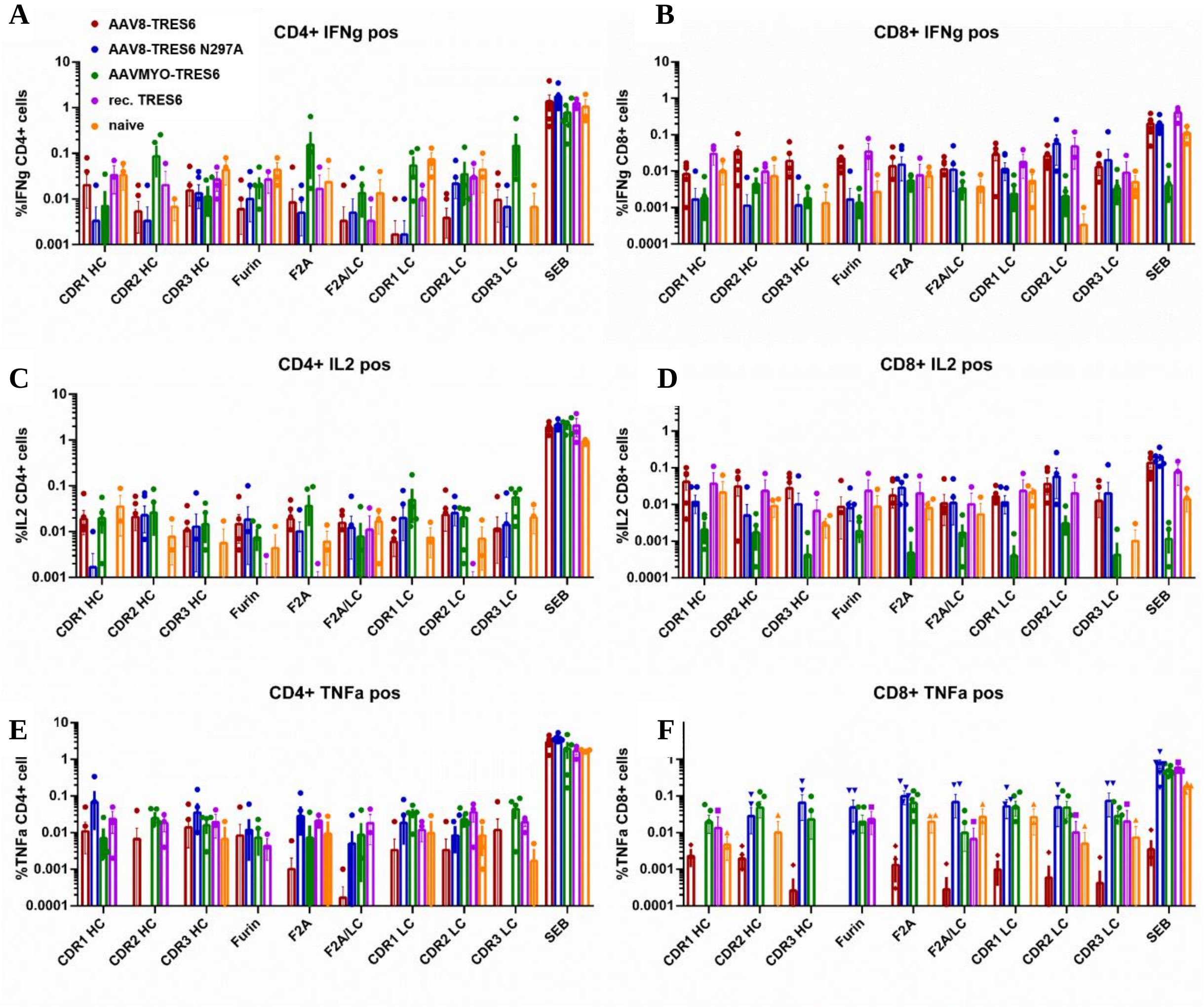

Figure S4. Anti-drug T cell responses. Splenocytes were obtained from mice 52 weeks after administration of AAV vector particles, recombinant TRES6 or naïve animals *(AAV vector group size n = 6, ♀ = 3, ♂ = 3; TRES6 and naïve group size n = 3, ♀ = 2, ♂ = 1)* and restimulated with peptides spanning antibody fragments with the highest immunogenic potential. Each dot represents results from a single mouse. The bars show for each group (red: AAV8-TRES6, blue: AAV8-TRES6 N297A, green: AAVMYO-TRES6, pink: recombinant TRES6, yellow: naïve) the mean percentage of CD4 positive cells that are IFNγ (A), IL2 (C) or TNFα (E) positive and of CD8 positive cells that are IFNγ (B), IL2 (D) or TNFα (F) positive. Background activation was determined by stimulation with a DMSO control for each mouse and subtracted. Statistical evaluation of the data was performed by an ordinary Two-way ANOVA test and Dunnett’s Pairwise Multiple Comparison Procedures as post hoc test in comparison to the naïve control (not indicated: non-significant).

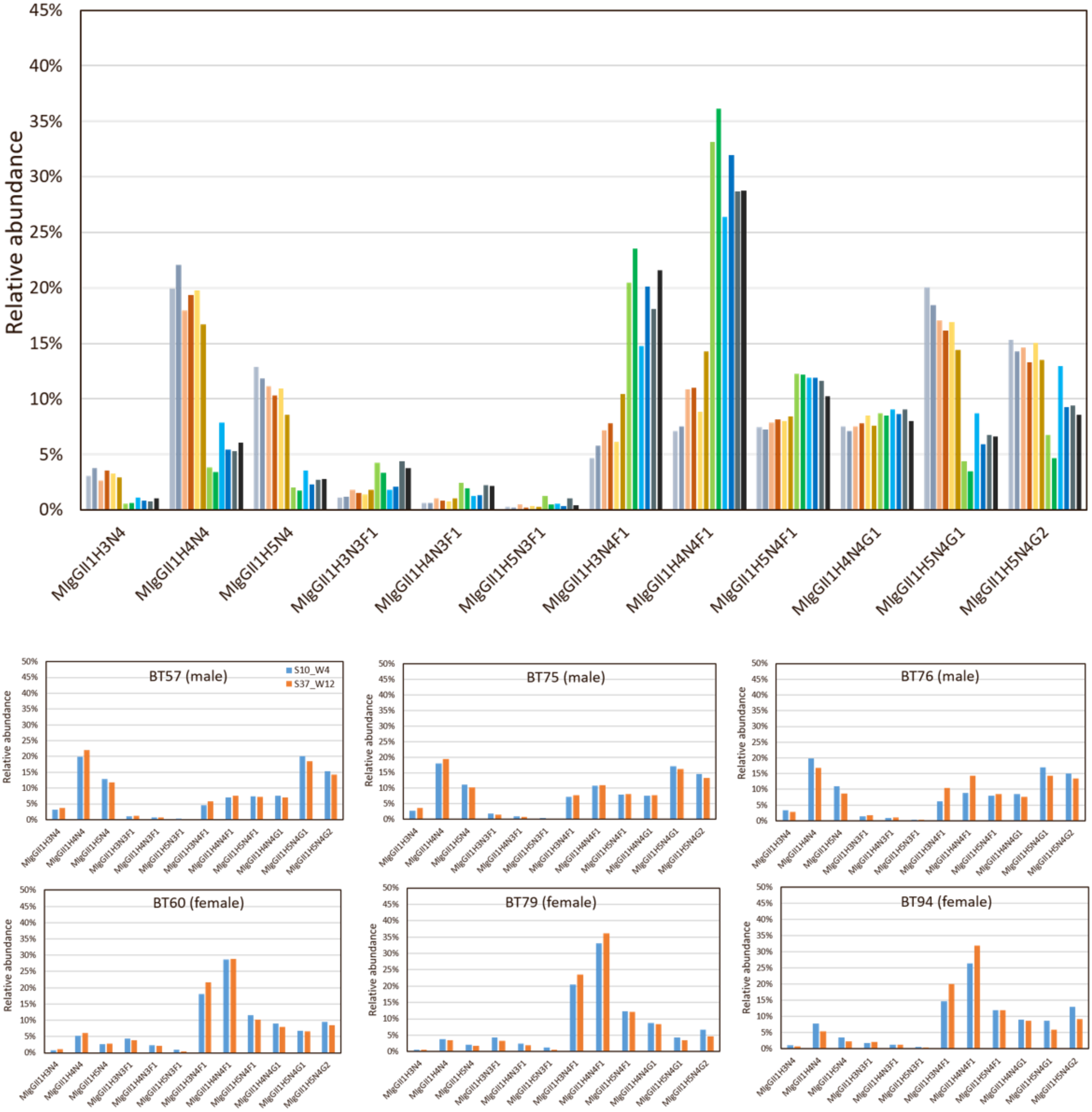

__Figure S5. Murine IgG2c Fc-glycosylation of AAV8 encoded antibodies.__ The relative abundance of different glycosylation profiles are shown for each mouse represented as color-coded bars. The proposed glycan structures are based on literature [63]. The matching light and strong colours show the value in week 4 and week 12, respectively, of the long-term experiment. Below, the glycosylation profile is shown for each mouse separately. Here, the blue bar represents week 4 and week 12 is shown in orange. H: hexose; N: N-acetylhexosamine; F: fucose; S: N-acetylneuraminic acid; G: N-glycolyneuraminic acid. Peptide sequence of IgG2a/c:EDYNSTLR. MIgGII: Murine IgG2c.

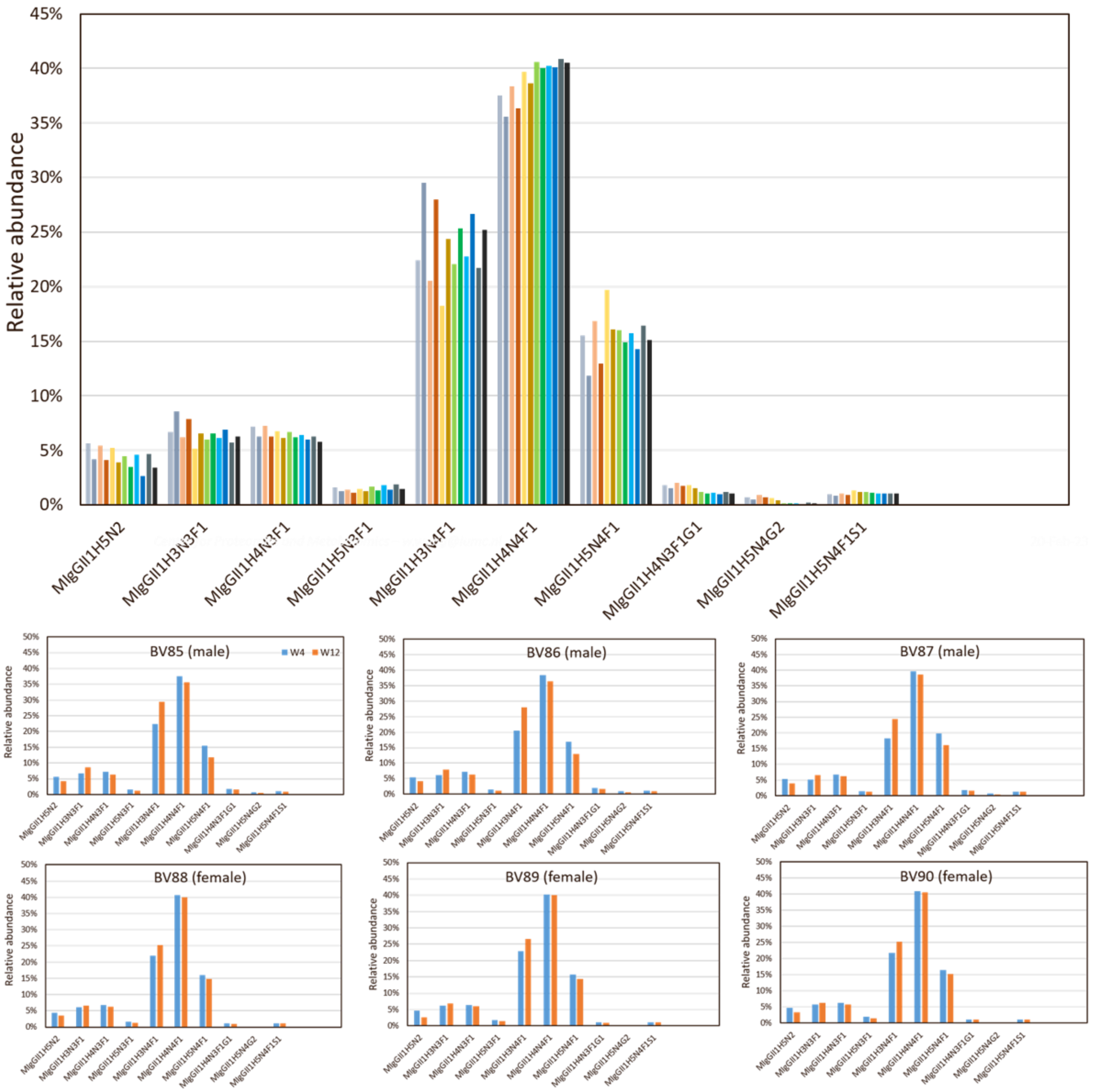

Figure S6 Murine IgG2c Fc-glycosylation of AAV8 encoded antibodies. *The relative abundance of different glycosylation profiles is shown for each mouse represented as a bar following AAV8 transduction. The glycan structure, colour code and abbreviations are described in Fig. S5 .*

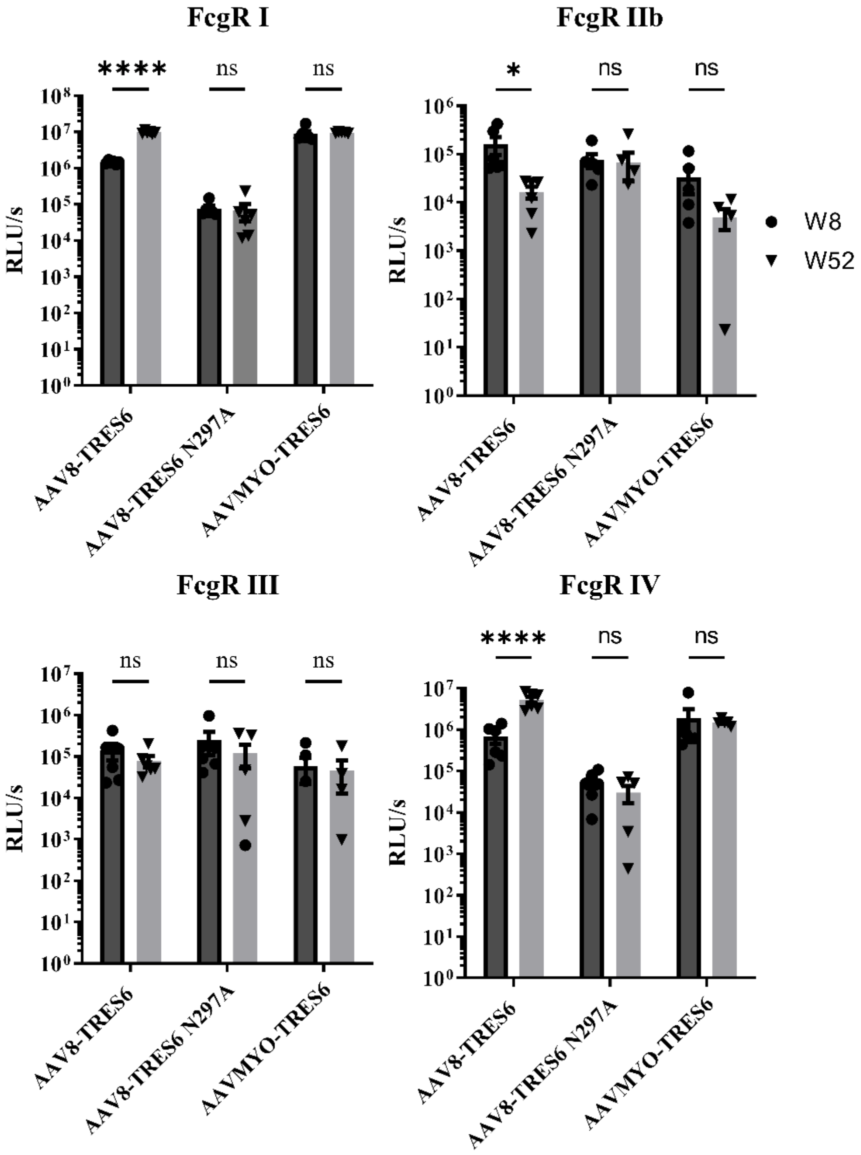

Figure S7. Changes in Fc-γ receptor binding over time. *The binding of serum TRES6 antibodies to the Fc-γ receptors RI - RIV was compared eight (black) and 52 (grey) weeks after injection of the indicated vector particles (group size n = 6, ♀ = 3, ♂ = 3). Statistical evaluation of the data was performed by Kruskal-Wallis test (one-way ANOVA) and Dunn's Pairwise Multiple Comparison Procedures as post-hoc test (*: $p \leq 0.01$; ****: $p \leq 0.0001$; ns: non-significant)*

Table S1. Transcriptomic expression analysis of genes associated with glycosylation pathways. *The transcriptome data of hepatocytes was compared between female and male C57BL/6 mice (Bioproject accessions PRJNA873076 (male C57BL/6J)*

and PRJNA893576 (female C57BL/6J)). The RNAseq data from tissue harvested at time point 6h [71] was analysed with CLC Genomic Workbench version 23 for differential expression in 2 groups (female vs male) with a focus on glycosylation pathway genes and was further condensed to N-Glycosylation related genes according to [58]. Differences, that were significant after Bonferroni-correction are highlighted in green.

	Name	Max group mean	Log fold change	Fold change	P-value	FDR p-value	Bonferroni	GeneID
Specific initiation	Stt3a	30,444	0,204	1,152	0,032	0,098	1	16430
	Uggt1	12,676	-0,228	-1,171	0,029	0,093	1	320011
	Uggt2	0,568	-0,019	-1,014	0,861	0,923	1	66435
Specific core extension	Mgat1	19,5941	0,2786	1,2130	1,65E-05	0,0002	0,4183	17308
	Mgat2	31,2620	-0,0691	-1,0490	0,4916	0,6630	1	217664
	Mgat3	0,0491	-0,0363	-1,0255	0,9160	0,9528	1	17309
	Mgat4a	0,2715	-0,0399	-1,0280	0,8012	0,8873	1	269181
	Mgat4b	31,4568	0,4864	1,4010	6,55E-11	2,58E-09	1,66E-06	103534
	Mgat4c	0,0028	1,7551	3,3754	0,0334	0,1021	1	67569
	Mgat5	2,1014	-0,3084	-1,2384	0,0041	0,0193	1	107895
	Fut8	3,4095	0,7054	1,6306	1,99E-10	7,17E-09	5,06E-06	53618
	Dpm1	20,4877	0,2721	1,2076	0,0051	0,0227	1	13480
	Dpagt1	20,5655	0,1840	1,1360	0,0144	0,0529	1	13478
	Alg13	1,9034	-0,0261	-1,0183	0,7897	0,8793	1	67574
	Alg14	11,9072	0,2675	1,2037	0,0001	0,0012	1	66789
	Alg1	20,8698	0,2630	1,2000	0,0001	0,0011	1	208211
	Alg2	9,8843	0,0005	1,0004	0,9964	0,9979	1	56737
	Alg11	9,6640	-0,1399	-1,1018	0,0406	0,1180	1	207958
	Alg12	5,6362	-0,0340	-1,0239	0,8219	0,9008	1	223774
	Alg3	2,7441	0,2450	1,1851	0,0077	0,0319	1	208624
	Alg9	6,8175	0,2603	1,1978	0,0003	0,0022	1	102580
	Alg6	4,4799	0,0671	1,0476	0,4945	0,6657	1	320438
	Alg8	2,4932	0,2011	1,1496	0,0351	0,1059	1	381903
	Alg10b	11,7276	-0,4610	-1,3765	0,0014	0,0080	1	380959
Non-specific Elonagation and branching	B3galt1	7,4117	-1,0744	-2,1058	3,22E-11	1,32E-09	8,18E-07	26877
	B3galt2	0,0526	1,9852	3,9593	0,0011	0,0065	1	26878
	B3galt5	0,0064	0,1707	1,1256	0,7395	0,8429	1	93961
	B4galnt3	0,6842	-0,7903	-1,7294	1,36E-05	0,0001	0,3445	330406
	B4galnt4	0,0824	-0,2320	-1,1745	0,5860	0,7347	1	330671
	B4galt1	14,6070	0,1953	1,1450	0,0186	0,0648	1	14595
	B4galt2	0,1430	0,1757	1,1295	0,5140	0,6818	1	53418
	B4galt3	7,5608	0,2810	1,2150	0,0009	0,0053	1	57370

	B4galt4	0,8635	0,1607	1,1178	0,2606	0,4425	1	56375
	B3gnt2	14,1687	-0,2142	-1,1600	0,0069	0,0293	1	53625
	B3gnt3	1,4823	0,2108	1,1574	0,1978	0,3685	1	72297
	B3gnt4	0,0463	0,2220	1,1663	0,6064	0,7517	1	231727
	B3gnt7	0,0527	1,3717	2,5878	0,0036	0,0173	1	227327
	B3gnt8	0,9237	0,3423	1,2678	0,0635	0,1653	1	232984
	B3gnt9	0,4543	0,5472	1,4613	0,0246	0,0808	1	97440
	Gcnt2	4,3058	0,1361	1,0990	0,1203	0,2619	1	14538
	Gcnt7	0,0370	-0,6004	-1,5161	0,1933	0,3627	1	654821
Non-specific - capping	*St3gal1*	35,5026	0,2975	1,2290	0,0120	0,0456	1	20442
	St3gal2	0,7200	0,3402	1,2659	0,0193	0,0668	1	20444
	St3gal3	34,3926	-0,2078	-1,1549	0,0156	0,0564	1	20441
	St3gal4	13,6150	0,2841	1,2176	0,0019	0,0103	1	20443
	St3gal5	121,7287	0,4852	1,3998	0,0106	0,0415	1	20454
	St3gal6	2,9770	2,1123	4,3239	2,91E-58	2,02E-55	7,40E-54	54613
	St6gal1	30,9089	0,4075	1,3264	0,0012	0,0067	1	20440
	St6gal2	0,0005	-0,0398	-1,0279	0,9606	#NV	1	240119
	St6galnac1	0,0027	0,9048	1,8723	0,2966	0,4767	1	20445
	St6galnac2	0,5702	0,4438	1,3602	0,0108	0,0420	1	20446
	St6galnac3	0,0677	-0,0643	-1,0456	0,7574	0,8561	1	20447
	St6galnac4	1,0375	1,0585	2,0827	3,68E-08	7,99E-07	0,0009	20448
	St6galnac5	0,0045	0,9071	1,8753	0,2936	0,4751	1	26938
	St6galnac6	31,5668	0,1761	1,1299	0,0755	0,1879	1	50935
	St8sia1	0,0261	-0,8568	-1,8110	0,0106	0,0415	1	20449
	St8sia2	0,0111	-0,1304	-1,0946	0,7738	0,8678	1	20450
	St8sia3	0,1751	0,2352	1,1771	0,3067	0,4874	1	20451
	St8sia4	0,2685	-0,1469	-1,1072	0,4313	0,6106	1	20452
	St8sia5	0,0457	1,9001	3,7325	0,0058	0,0253	1	225742
	St8sia6	0,0139	0,7018	1,6265	0,1524	0,3098	1	241230
	B4galnt2	0,2328	-1,2107	-2,3144	0,0053	0,0236	1	14422
	A4gnt	0,0284	-0,8285	-1,7758	0,1358	0,2860	1	333424
	Abo	0,0008	0,3042	1,2347	0,7442	#NV	1	80908
	B3gat1	0,0107	0,0451	1,0318	0,9284	0,9604	1	76898
	B3gat2	0,1680	-0,2887	-1,2215	0,2669	0,4498	1	280645
	Fut1	0,0642	-0,0016	-1,0011	0,9970	0,9981	1	14343
	Fut2	0,0573	-0,6928	-1,6164	0,1051	0,2382	1	14344
	Fut4	0,1065	-0,3003	-1,2314	0,3535	0,5362	1	14345
	Fut7	0,0605	0,4795	1,3942	0,2403	0,4209	1	14347
	Fut9	0,0015	0,6553	1,5749	0,3327	0,5158	1	14348
	Fut10	0,1522	0,2088	1,1557	0,2744	0,4572	1	171167
	Fut11	2,3442	-0,0367	-1,0258	0,7611	0,8584	1	73068

Non-specific - Sulfation	*Gal3st1*	*1,6974*	*0,0572*	*1,0404*	*0,8224*	*0,9011*	*1*	[*53897*]
	Chst8	*0,0422*	*0,4904*	*1,4048*	*0,2458*	*0,4279*	*1*	[*68947*]
	Chst9	*0,0254*	*0,9270*	*1,9014*	*0,0860*	*0,2062*	*1*	[*71367*]
	Gal3st3	*0,0068*	*0,1366*	*1,0993*	*0,8327*	*0,9085*	*1*	[*545276*]
	Gal3st2	*0,1103*	*-0,9146*	*-1,8850*	*0,0888*	*0,2108*	*1*	[*381334*]
	Gal3st4	*0,1266*	*0,3962*	*1,3160*	*0,1248*	*0,2689*	*1*	[*330217*]
	Chst1	*0,1025*	*1,0561*	*2,0792*	*0,0035*	*0,0167*	*1*	[*76969*]
	Chst3	*0,0499*	*0,3853*	*1,3061*	*0,2705*	*0,4532*	*1*	[*53374*]
	Chst5	*0,0000*	*#NV*	*#NV*	*#NV*	*#NV*	*#NV*	[*56773*]
	Chst7	*0,5739*	*-0,0406*	*-1,0285*	*0,8336*	*0,9089*	*1*	[*60322*]
	Chst4	*0,2439*	*0,9542*	*1,9375*	*0,0223*	*0,0748*	*1*	[*26887*]
	Chst2	*0,9032*	*0,4014*	*1,3208*	*0,0117*	*0,0448*	*1*	[*54371*]
	Chst10	*0,0709*	*0,8260*	*1,7728*	*0,0352*	*0,1061*	*1*	[*98388*]

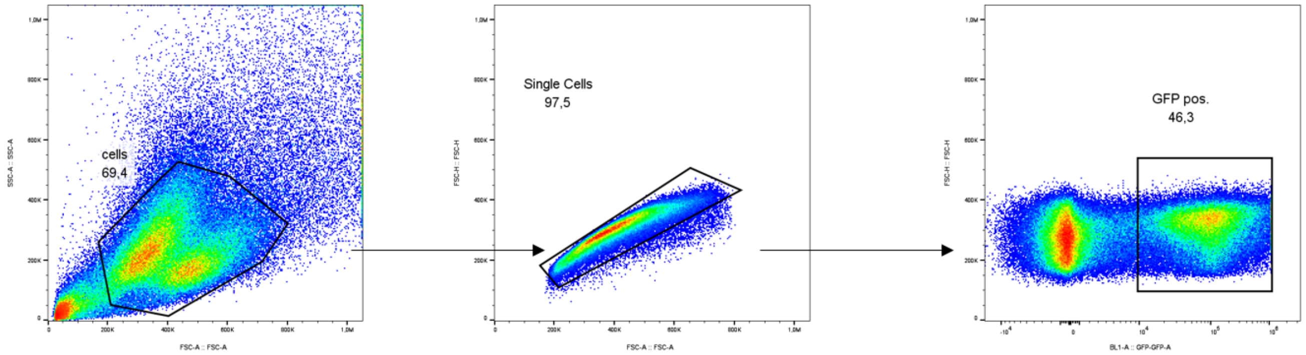

Figure S8. Gating strategy for antibody binding. *Shown is the gating strategy for the analysis of antibody binding to Spike expressing cells.*

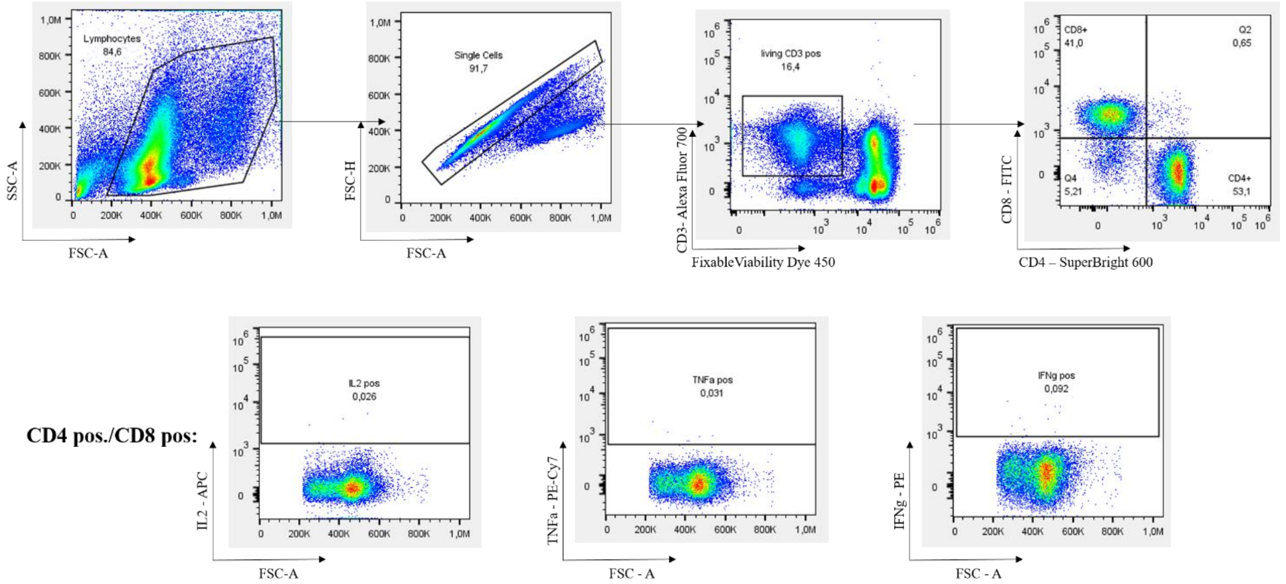

Figure S9. Gating strategy for the analysis of anti-drug T cell responses. *Shown is the gating strategy for the identification of CD4+ and CD8+ T cells. Within these populations, the frequency of IL2 positive, TNFα positive or IFNγ positive cells was determined.*
